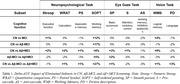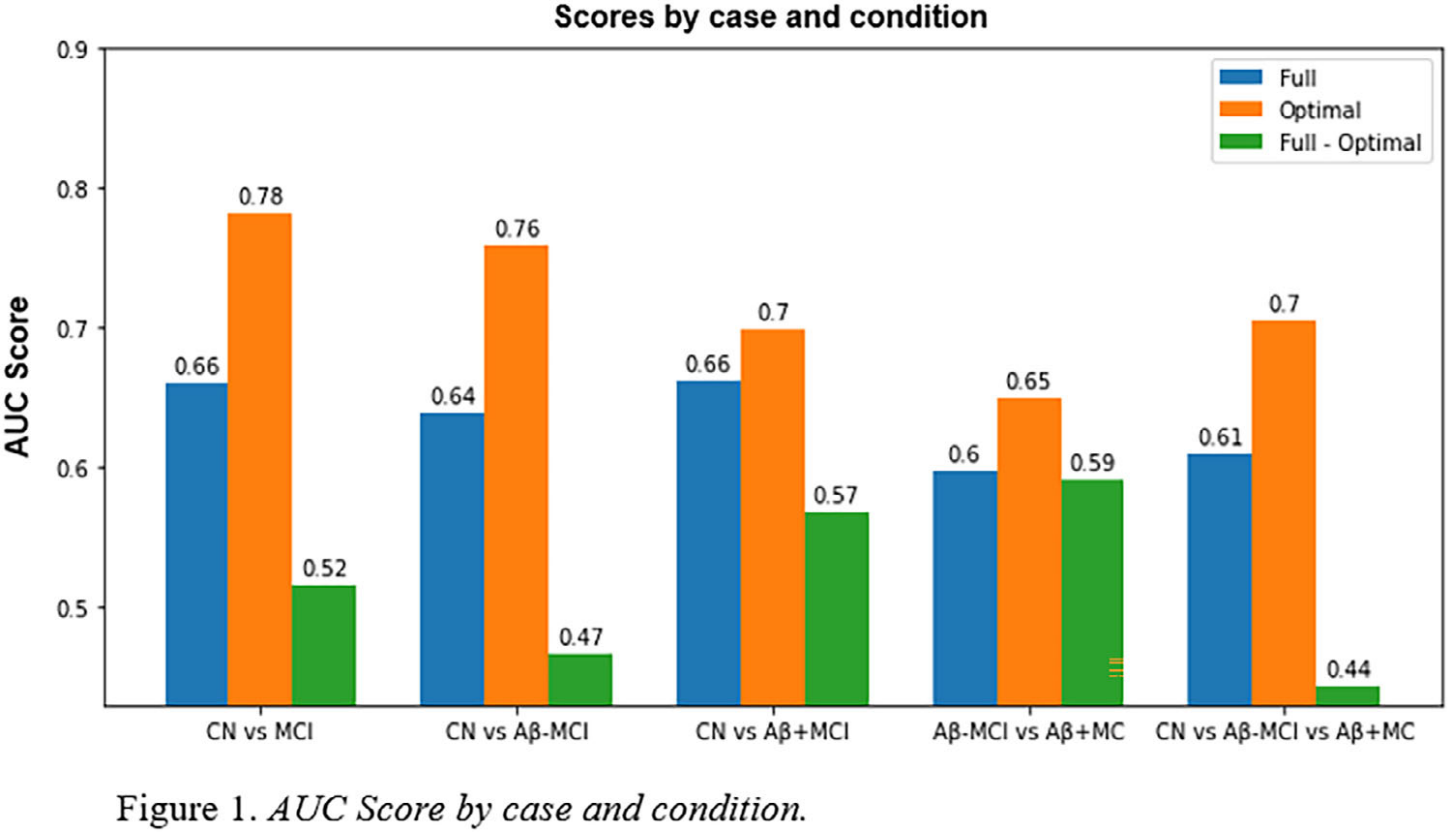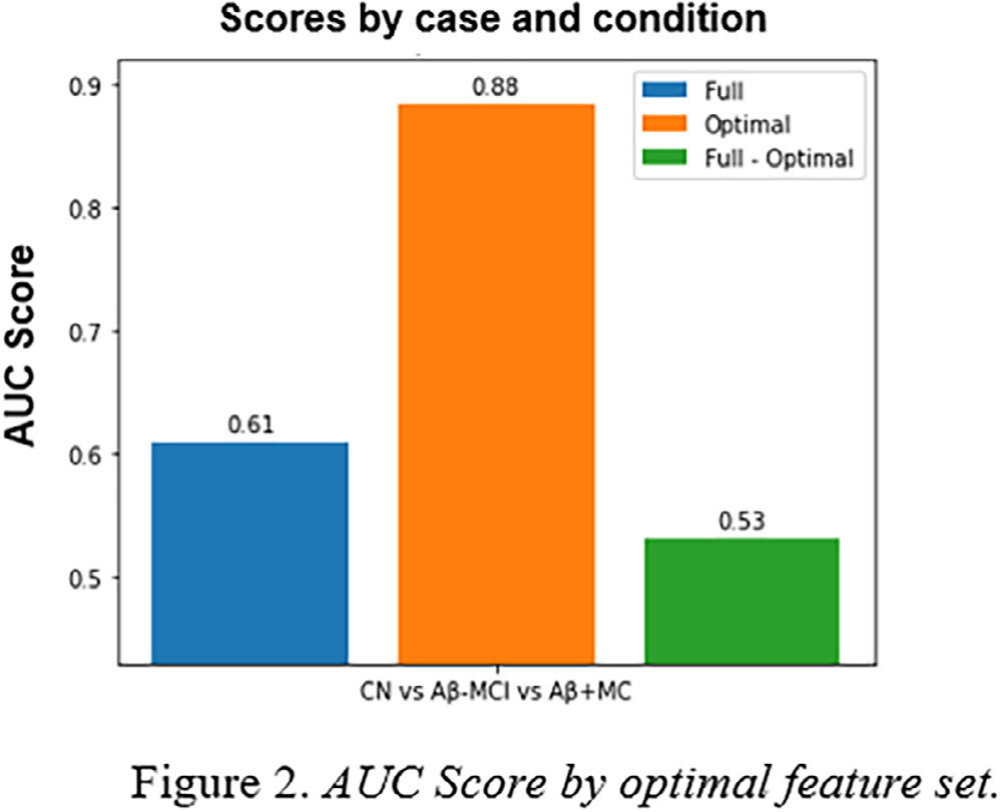# Investigating the Validity of Digital Biomarkers in the Preclinical Screening of Cognitive Normal, Amyloid‐β‐ MCI and Amyloid‐β+ MCI

**DOI:** 10.1002/alz.090074

**Published:** 2025-01-09

**Authors:** Whani Kim, Jin Sung Kim, Hyun Jeong Ko, Byung Hun Yun, Yuyoung Kim, Dong Han Kim, Ui Jun Kwon, Sang Kwon Lim, Bo Ri Kim, Jee Hang Lee, Geon Ha Kim, Jinwoo Kim

**Affiliations:** ^1^ HAII Inc., Seoul Korea, Republic of (South); ^2^ Sangmyung University, Seoul Korea, Republic of (South); ^3^ Yonsei University, Seoul Korea, Republic of (South); ^4^ Ewha Womans University, Seoul Korea, Republic of (South); ^5^ Ewha Womans University Mokdong Hospital, Ewha Womans University College of Medicine, Seoul Korea, Republic of (South); ^6^ Ewha Brain Institute, Ewha Womans University, Seoul Korea, Republic of (South)

## Abstract

**Background:**

Amyloid‐β is widely known as a substantial biomarker in the diagnosis of Alzheimer’s disease. However, detection of amyloid‐β through neuroimaging techniques requires huge amounts of resources. There is a growing demand to detect these pathologies based on digital biomarkers. This study primarily aimed to examine the validity of a mobile‐based tool for assessment of cognitive impairment (mACI) in the prediction of cognitive normal (CN), amyloid‐β negative MCI (Aβ‐MCI), and amyloid‐β positive MCI (Aβ+MCI).

**Methods:**

We recruited 102 participants from Gwangju Alzheimer’s and Related Dementia (GARD) cohort containing K‐MMSE v2, SNSB v2, MRI, PET‐CT, and clinical diagnosis data. 48 participants, who were still in the process of receiving a clinical diagnosis, were excluded. We then collected mACI data, which examined nine cognitive functions proposed in Kim et al. (2023), resulting in 54 participants’ full dataset. Medical doctors labeled the participants into CN (*n* = 27), Aβ‐MCI (*n* = 14), and Aβ+MCI (*n* = 13). We subsequently analyzed the data using machine learning (ML) techniques to see whether the mACI is a successful tool for preclinical screening between them.

**Results:**

We built a predictive model to classify the participants into CN, Aβ‐MCI and Aβ+MCI using the Catboost ML algorithm. To see the result, we performed *Recursive Subtest Elimination Cross Validation* (RSECV). In doing so, we measured the delta‐AUC indicating the degree of contribution of the eliminated subtest in the preclinical screening of CN, Aβ‐MCI and Aβ+MCI. As shown in Table 1, there are clearly distinctive sets of subtests when screening the participants into CN, Aβ‐MCI and Aβ+MCI. Specifically, the subtest in relation to working memory is likely to be crucial in classifying CN and MCI, and the additional subtest related to executive function plays an important role in classifying Aβ+MCI and others.

**Conclusion:**

In this study, we found that mACI was able to successfully classify CN, Aβ‐MCI and Aβ+MCI. Our work serves as foundational work to reduce screening time and expenses to identify amyloid‐β accumulation using a mobile‐based application.